# Geographical variation in HIV testing in South Africa: Evidence from the 2017 national household HIV survey

**DOI:** 10.4102/sajhivmed.v22i1.1273

**Published:** 2021-08-31

**Authors:** Sean Jooste, Musawenkosi Mabaso, Myra Taylor, Alicia North, Yolande Shean, Leickness C. Simbayi, Tarylee Reddy, Leonard Mwandingi, Tenielle Schmidt, Portia Nevhungoni, Samuel Manda, Khangelani Zuma

**Affiliations:** 1 Human Sciences Research Council, Cape Town, South Africa; 2 School of Nursing and Public Health, University of KwaZulu-Natal, Durban, South Africa; 3 Human Sciences Research Council, Durban, South Africa; 4 Department of Psychiatry & Mental Health, University of Cape Town, Cape Town, South Africa; 5 South African Medical Research Council, Durban, South Africa; 6 Division of Epidemiology and Biostatistics, Stellenbosch University, Cape Town, South Africa; 7 Ministry of Health and Social Sciences, Windhoek, Namibia; 8 South African Medical Research Council, Pretoria, South Africa; 9 Department of Statistics, University of Pretoria, Pretoria, South Africa; 10 Human Sciences Research Council, Pretoria, South Africa; 11 School of Public Health, University of the Witwatersrand, Johannesburg, South Africa

**Keywords:** HIV, HIV testing, thematic mapping, districts, South African

## Abstract

**Background:**

Identification of the geographical areas with low uptake of HIV testing could assist in spatial targeting of interventions to improve the uptake of HIV testing.

**Objectives:**

The objective of this research study was to map the uptake of HIV testing at the district level in South Africa.

**Method:**

The secondary analysis used data from the Human Sciences Research Council’s 2017 National HIV Prevalence, Incidence, Behaviour and Communication Survey, where data were collected using a multistage stratified random cluster sampling approach. Descriptive spatial methods were used to assess disparities in the proportion of those ever tested for HIV at the district level in South Africa.

**Results:**

The districts with the highest overall coverage of people ever having tested for HIV (> 85%) include West Rand in Gauteng, Lejweleputswa and Thabo Mofutsanyane in Free State, and Ngaka Modiri Molema in North-West. These provinces also had the least variation in HIV testing coverage between their districts. Districts in KwaZulu-Natal had the widest variation in coverage of HIV testing. The districts with the lowest uptake of HIV testing were uMkhanyakude (54.7%) and Ugu (61.4%) in KwaZulu-Natal and Vhembe (61.0%) in Limpopo. Most districts had a higher uptake of HIV testing amongst female than male participants.

**Conclusion:**

The uptake of HIV testing across various districts in South Africa seems to be unequal. Intervention programmes must improve the overall uptake of HIV testing, especially in uMkhanyakude and Ugu in KwaZulu-Natal and Vhembe in Limpopo. Interventions must also focus on enhancing uptake of HIV testing amongst male participants in most districts. Strategies that would improve the uptake of HIV testing include HIV self-testing and community HIV testing, specifically home-based testing.

## Introduction

Eastern and Southern Africa is home to 53% of the 36.9 million people living with HIV globally,^1^ with an estimated 75% of people living with HIV who actually knew their HIV status by the end of 2017.^1^

Furthermore, there was a 42% reduction of AIDS-related illnesses, as a result of the increase in HIV testing and treatment coverage between 2010 and 2017.^1^

South Africa has one of the largest HIV testing services (HTS), which is a crucial component of national HIV response.^2^ HIV testing services are vital in directing HIV-positive people to the treatment continuum, starting with antiretroviral therapy and, therefore, is critical in the fight against HIV.^2^ The Joint United Nations Programme on HIV (UNAIDS) launched the 90-90-90 targets stipulating that by 2020, 90% of people living with HIV should know their status, 90% of those who know their HIV-positive status should receive antiretroviral therapy and 90% of those on treatment have a suppressed viral load to end the epidemic by 2030. The UNAIDS has revised 2030 targets of 95-95-95, which are set out to be achieved by 2030.^3^

South Africa has made progress towards the UNAIDS 90-90-90 targets, especially regarding HIV testing and viral load suppression.^4^ Over the past decade, the country had made excellent progress in involving more people to test and become aware of their HIV status, after the launch of two national HIV testing initiatives: firstly, the national HIV testing and counselling (HTC) campaign that took place in 2010, and secondly, the HTC revitalisation strategy in 2013.^5^ As a result of these campaigns and other similar campaigns, more than 10 million people in South Africa test for HIV every year.^5^ In scaling up efforts around HTS interventions, civil society organisations continue to work with government departments in South Africa. The South African National AIDS Council continues to provide a platform for engagement between the civil society and government to work together on the HIV response.^6^

Although South Africa has made steady progress towards reaching the UNAIDS targets, many people affected with HIV are still unaware of their HIV status.^7^ Despite the availability of HTS, research studies have revealed that only a fraction of South Africans who are at risk get tested for HIV.^8^ Evidence shows that access to HTS may be limited geographically because of the inadequacy and heterogeneous distribution of available services.^9,10^ Achieving high coverage of HIV testing is critical for linking HIV-positive people to care across the country. Therefore, equitable geographical distribution of HTS is vital for achieving optimal coverage for HIV testing.^10^ This highlights the importance of conducting and collecting population-based HIV testing covereage data at the sub-national level needed for decision making.

In South Africa, gathering spatial data on HIV and mapping its distribution have been carried out in selected micro-geographical areas, limiting the generalisability of the findings to the country.^11^ The main source of estimating the number of people who tested for HIV in the country comes from the District Health Information System and from modelling.^12^ Both sources have limitations and rely on healthcare facility and programme data from districts to produce estimates.^13,14^ The current study used large-scale nationally representative population-based household survey data to describe the spatial coverage in the uptake of HIV testing amongst youth and adults 15 years and older. The aim of this research study was to identify the spatial gap in the uptake of testing in people who had ever tested for HIV at the district level in South Africa.

## Methods

### Study design and sampling

The data used in the secondary analysis were obtained from the National HIV Prevalence, Incidence, Behaviour and Communication Survey conducted in 2017.^15^ The survey used a multistage stratified, cluster randomised, cross-sectional design. The survey chose a systematic probability sample of 15 households randomly from 1000 small area layers (SALs), selected from 84 907 SALs released by Statistics South Africa in 2015.^16^ The sampling of SALs was stratified by province and locality type (urban formal, urban informal, rural formal and rural informal localities). An additional 457 SALs were sampled in 13 high-priority districts, which included iLembe, uMzinyathi, uThukela and King Cetshwayo in KwaZulu-Natal province; Ehlanzeni and Gert Sibande in Mpumalanga province; O.R. Tambo in the Eastern Cape province; Sekhukhune in Limpopo province; Bojanala Platinum in North-West province; and Ekurhuleni, Sedibeng, Tshwane and West Rand in Gauteng province. This study focused on the population aged 15 years and older who reported ever testing for HIV.

### Measures

The primary outcome measure ‘ever testing for HIV’ was obtained from individuals who responded to the original survey question ‘have you ever been tested for HIV?’ The response was dichotomised into a binary outcome (yes = 1 and no = 0).

### Ethical considerations

The survey protocol was approved by the Human Sciences Research Council’s (HSRC) Research Ethics Committee (REC: 4/18/11/15), and the Associate Director for Science, Center for Global Health, Centres for Disease Control and Prevention (CDC). Ethical clearance was also obtained from the University of KwaZulu-Natal’s Biomedical Research Ethics Committee (BE 646/18). Verbal or written informed consent was sought before undertaking both the behavioural data and blood specimen collection.

### Statistical analysis

Statistical analysis was carried out in STATA 15.0 (Stata Corporation, College Station, TX, United States [US]) software.

Descriptive statistics were used to summarise the sample characteristics. Multilevel mixed-effects logistic regression models were used to estimate the excess probability of prior testing for HIV after adjusting for the effect of age and sex. District-level random effects predicted from the model, including age and sex were used to estimate the excess probability of prior testing. Results are shown with 95% confidence intervals (CI), and p-values < 0.05 were reported for all statistically significant associations. The proportion of the population, aged 15 years and older, that have ever been tested for HIV were geo-located using the South African district-level boundaries. The maps were generated in QGIS, version 3.14.10. An adjusted weight, benchmarked to the general population by age and sex at the national level, was computed to facilitate this analysis.

### Results

#### Socio-demographic characteristics of the study sample

Table 1 shows the mean age and sex distribution amongst the respondents in all 52 districts. uMkhanyakude, King Cetshwayo (both in KwaZulu-Natal) and Gert Sibande in Mpumalanga had the youngest mean age of under 35 years. Amathole in the Eastern Cape, Fezile Dabi in Free State and Namakwa in the Northern Cape had the oldest mean age of 43 years. Harry Gwala, uThukela, uMzinyathi (all in KwaZulu-Natal) and Buffalo City in the Eastern Cape had the highest proportion of female participants (over 59%). Fezile Dabi in Free State, West Coast in Western Cape, Nkangala in Mpumalanga and uMgungundlovu in KwaZulu-Natal had the highest proportion of male participants (over 54%).

**TABLE 1:** Mean age and sex distribution of youth and adult 15 years and older by district, South Africa 2017.

Province	District name	*n*	Mean age (years)	Male (*%*)	Female (*%*)
Eastern Cape	Alfred Nzo	278	40.2	42.0	58.0
Eastern Cape	Amathole	337	44.6	46.4	53.6
Eastern Cape	Buffalo City	329	41.7	40.9	59.1
Eastern Cape	Chris Hani	243	42.0	47.9	52.1
Eastern Cape	Joe Gqabi	188	41.1	53.1	46.9
Eastern Cape	Nelson Mandela Bay	1213	41.9	48.6	51.4
Eastern Cape	O.R. Tambo	1369	39.9	45.3	54.7
Eastern Cape	Sarah Baartman	712	39.8	49.0	51.0
Free State	Fezile Dabi	263	44.2	55.8	44.2
Free State	Lejweleputswa	365	39.0	49.0	51.0
Free State	Mangaung	1068	39.7	48.9	51.1
Free State	Thabo Mofutsanyane	776	39.2	48.7	51.3
Free State	Xhariep	243	39.2	52.6	47.4
Gauteng	City of Johannesburg	1754	40.0	49.4	50.6
Gauteng	City of Tshwane	1718	38.9	50.3	49.7
Gauteng	Ekurhuleni	2011	38.0	51.6	48.4
Gauteng	Sedibeng	2894	39.1	50.9	49.1
Gauteng	West Rand	1192	38.2	52.5	47.5
KwaZulu-Natal	Amajuba	287	41.4	41.5	58.5
KwaZulu-Natal	eThekwini	3583	41.7	47.3	52.7
KwaZulu-Natal	Harry Gwala	427	37.7	38.6	61.4
KwaZulu-Natal	iLembe	3605	36.0	44.2	55.8
KwaZulu-Natal	King Cetshwayo	4003	34.3	43.9	56.1
KwaZulu-Natal	Ugu	958	40.0	48.1	51.9
KwaZulu-Natal	uMgungundlovu	601	41.2	54.6	45.4
KwaZulu-Natal	uMkhanyakude	651	33.5	41.0	59.0
KwaZulu-Natal	uMzinyathi	3227	37.5	40.6	59.4
KwaZulu-Natal	uThukela	3770	36.4	40.1	59.9
KwaZulu-Natal	Zululand	480	37.5	46.0	54.0
Limpopo	Capricorn	659	40.0	42.2	57.8
Limpopo	Greater Sekhukhune	1292	39.2	42.9	57.1
Limpopo	Mopani	604	41.2	46.3	53.7
Limpopo	Vhembe	705	37.8	47.7	52.3
Limpopo	Waterberg	480	40.7	53.1	46.9
Mpumalanga	Ehlanzeni	2731	35.3	47.5	52.5
Mpumalanga	Gert Sibande	3585	34.4	52.2	47.8
Mpumalanga	Nkangala	1247	36.7	54.9	45.1
North West	Bojanala	2322	37.3	48.5	51.5
North West	Dr Kenneth Kaunda	761	37.2	51.9	48.1
North West	Dr Ruth Segomotsi Mompati	372	39.6	43.4	56.6
North West	Ngaka Modiri Molema	447	39.1	48.0	52.0
Northern Cape	Frances Baard	749	38.7	50.6	49.4
Northern Cape	John Taolo Gaetsewe	262	36.7	50.3	49.7
Northern Cape	Namakwa	200	43.3	50.5	49.5
Northern Cape	Pixley ka Seme	1005	37.2	49.2	50.8
Northern Cape	Z F Mgcawu	830	37.2	50.7	49.3
Western Cape	Cape Winelands	750	40.9	45.7	54.3
Western Cape	Central Karoo	108	42.3	44.0	56.0
Western Cape	City of Cape Town	2362	38.5	49.9	50.1
Western Cape	Eden	374	39.5	51.7	48.3
Western Cape	Overberg	305	40.9	46.8	53.2
Western Cape	West Coast	468	36.4	55.8	44.2

#### District-level coverage of ever being tested for HIV

**FIGURE 1:**
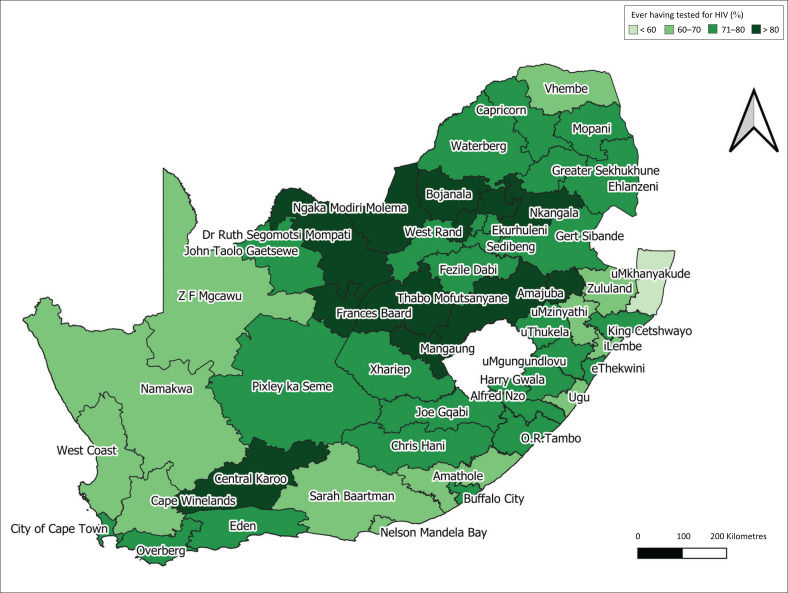
Geographical uptake of those aged 15 years and older who have ever been tested for HIV in the 52 districts of South Africa.

Figure 1 illustrates the geographical distribution of people who have ever been tested for HIV in the 52 districts of South Africa (Table 1-A1). The overall HIV testing uptake range was between 54.7% and 86.1%. Free State and North-West had more districts with an HIV testing coverage of over 80%, while no district in the Eastern Cape or Limpopo had an overall coverage higher than 80%.

Overall, uMkhanyakude (54.7%), Vhembe (61.0%) and Ugu (61.4%) districts had the lowest coverage for HIV testing. Ngaka Modiri Molema district (86.1%) reported the highest coverage for testing, followed by Lejweleputswa (85.2%) and Thabo Mofutsanyane (84.8%) district.

In the Eastern Cape, Joe Gqabi district had the highest overall coverage (78.5%), while Sarah Baartman district had the lowest (66.2%) coverage for HIV testing.

In the Free State, Lejweleputswa district had the highest testing uptake, followed by Thabo Mofutsanyane district, while Xhariep district (73.0%) had the lowest. In Gauteng, West Rand district had the highest coverage (83.3%), and the City of Johannesburg had the lowest coverage for testing (78.2%).

In KwaZulu-Natal, Amajuba district (83.1%) had the highest coverage, followed by Ugu district (61.4%), while uMkhanyakude district (54.7%) had the lowest coverage in the country. KwaZulu-Natal was the only province with a significant difference in testing coverage between its districts (*P* < 0.001).

In Limpopo, Waterberg district had the highest overall coverage (75.9%), while Vhembe district (61.1%) had the lowest coverage for testing. In Mpumalanga, Nkangala district had the highest overall coverage (80.4%), while Gert Sibande district (74.3%) had the lowest coverage for HIV testing. In North West, Ngaka Modiri Molema district (88.6%) had the highest coverage, while Dr Kenneth Kaunda district (76.9%) had the lowest coverage for testing. In the Northern Cape, Namakwa district (67.2%) had the lowest coverage, while Frances Baard district (81.4%) had the highest coverage.

In the Western Cape, Central Karoo district was the only district with over 80% coverage. In comparison, the West Coast and Cape Winelands district had the lowest coverage (< 70%), while the remaining districts’ coverage ranged from 70% to 79%.

#### District-level coverage of ever being tested for HIV by sex

**FIGURE 2:**
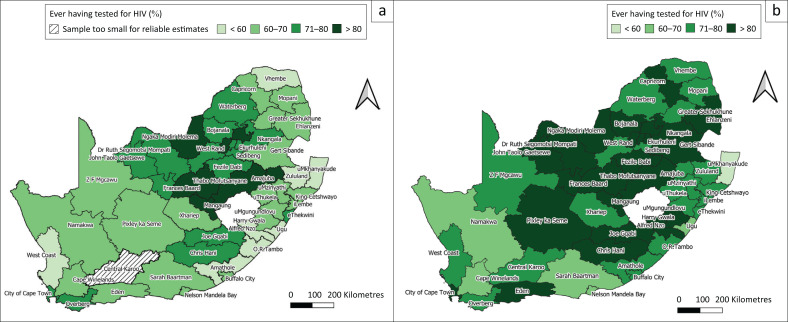
Geographical coverage: proportion of people who have ever been tested for HIV amongst (a) male and (b) female participants aged 15 years and older in the 52 districts in South Africa.

Figure 2 illustrates the geographical coverage of those who have ever been tested for HIV (Table 2-A1). The results are revealed for (1) male and (2) female participants, aged 15 years and older, across the 52 districts in South Africa. Overall, the maps show that female participants had coverage of over 80% in more districts than male participants. Female participants had a higher HIV testing rate of 20% more than male participants in Vhembe district (73.7% vs 46.6%), Eden (81.3% vs 61.1%), Alfred Nzo (85.5% vs 58.9%) and O.R. Tambo districts (79.6% vs 59.2%).

The proportion of female participants who had ever been tested for HIV ranged from 59.0% to 88.6%. uMkhanyakude district had the lowest proportion of female participants who had ever been tested for HIV (59.0%), followed by Ugu district (63.3%). Districts with the highest coverage of female participants who had ever been tested for HIV included Ngaka Modiri Molema (88.6%), Frances Baard (88.4%) and Lejweleputswa (88.4%). The coverage range of male participants who have ever been tested for HIV was 46.6% – 89.9%. Vhembe and uMkhanyakude were the only districts with < 50% coverage, that is, at 46.6% and 48.5%, respectively. Amajuba had the highest coverage (89.9%) of male participants who have ever been tested for HIV.

#### Adjusted coverage of ever being tested for HIV

**FIGURE 3:**
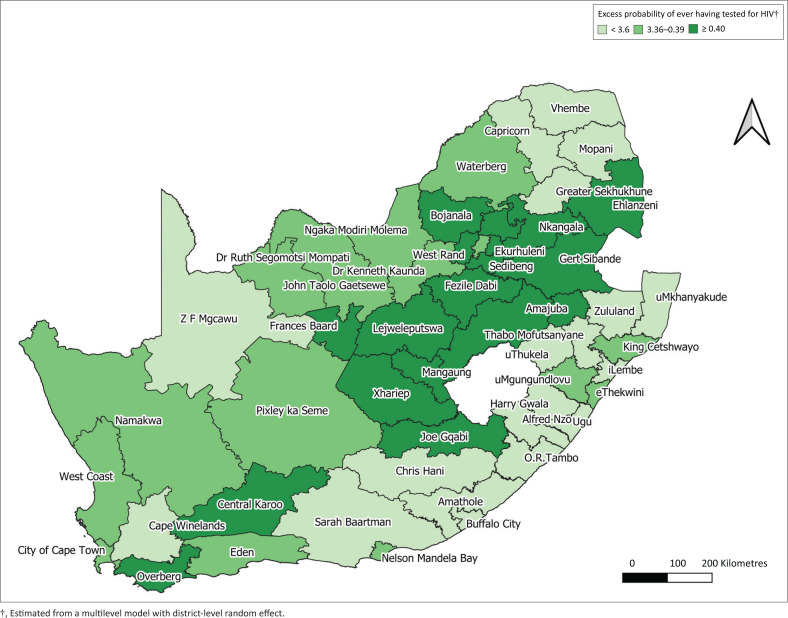
Geographical coverage of excess probability of ever having tested for HIV after adjusting for age and sex in the 52 districts in South Africa.

Figure 3 illustrates the geographical coverage of the excess probability of ever having tested for HIV in the 52 districts of South Africa after adjusting for age and sex (Table 3-A1). Both age and sex were significantly associated with previous testing (Table 4-A1). Specifically, female participants had a significantly higher odds of testing (OR: 1.59; 95% CI: 1.51–1.66) and a 1-year higher age associated with a 0.4% increase in the odds of testing. After adjusting for age and sex, the excess probability of ever having tested for HIV was different amongst the districts, illustrating that true heterogeneity (explained by variables other than sex and age) between the districts is present. The districts in Free State still had the highest probability for testing. Nkangala district had the second-highest probability for HIV testing. uMkhanyakude, Ugu, uMzinyathi, O.R. Tambo, Amathole, Chris Hani, Buffalo City, Vhembe and Greater Sekhukhune had the lowest probability for HIV testing.

## Discussion

HIV testing is a crucial component of the national HIV response in South Africa.^17^ This study presents the first geographic analysis of youth and adults (≥ 15 years) who have ever been tested for HIV in South Africa using simple GIS mapping and data obtained from a cross-sectional nationally representative population-based survey.

The mapping results revealed that the uptake of HIV testing varied across the various districts in South Africa. The age and sex distribution across the districts were different. Studies have revealed that age and sex are crucial factors in HIV testing.^18,19^ The estimates from a multilevel model with district-level random effects showed that excess probability of ever having tested for HIV was different among the districts after adjusting for age and sex. Variations in the quality of healthcare services, health promotion activities, easier access to healthcare facilities and socio-economic status could have an impact on the uptake of HIV testing in districts.

The overall proportion of people who had ever tested for HIV at the district level in South Africa ranged from 54.7% to 86.1%. uMkhanyakude and Ugu districts in KwaZulu-Natal and Vhembe district in Limpopo had the lowest overall testing coverage of < 62%. Ngaka Modiri Molema district in North West, and Lejwelepuswa and Thabo Mofutsanyane districts both in Free State reported the highest coverage for HIV testing. None of the districts in the Eastern Cape or Limpopo had an overall coverage of higher than 80%. These districts are characterised as being predominately rural. Other studies have also found that people living in rural informal or tribal areas were significantly less likely to test for HIV when compared with those from urban areas.^20,21^ The finding that uptake of HIV testing was less likely amongst those in rural areas could be linked to limited resources and structural barriers to healthcare in terms of geographical and financial accessibility.^22,23^ Additional barriers included fear, discrimination and stigmatising attitudes, as well as lack of education and awareness.^24^

Another factor playing a major role in the higher coverage districts included the epidemic control plans implemented by the President’s Emergency Plan for AIDS Relief (PEPFAR), which aims to achieve maximum impact and reach in areas with the highest burden of disease (COP19). This is informed by population-based surveillance. The PEPFAR country operational plan (COP) for 2017, in 27 districts with an estimated number of people living with HIV of 5.6 million, which account for 79% of number of people living with HIV in South Africa (COP19), identified 1969 sites for intensified support as part of the country’s district-level implementation plan (DIP).^25^ According to the National Strategic Plan for 2012–2016,^26^ the objectives included maximising opportunities for testing and screening to ensure that everyone in South Africa got tested for HIV and was screened for TB. The overall investment for HTS programmes in 2016–2017 was $126 663 865.00, with the South African Government funding being 45% and PEPFAR funding 55% (COP-19).

Most districts had a higher coverage of ever having tested for HIV amongst female than male participants. uMkhanyakude and Ugu had the lowest coverage for female participants. Vhembe and uMkhanyakude had the lowest HIV testing coverage for male participants. Despite the countrywide scale-up, the observed geographic disparities in HIV testing are relevant from an epidemic control perspective, especially if the people who do not get tested are at higher risk of HIV infection.^6^ Therefore, achieving high coverage of HIV testing amongst men is critical in the fight against HIV in the country. However, data elsewhere suggest that boys and men are lagging.^27,28^ Men were found to have lower levels of participation in HIV testing.^29^ Some of these reasons include fear of damaging reputations, losing their masculine pride, fearing both community rejection and a loss of emotional control because of the psychological burden of knowing one was HIV positive.^29^ HIV testing programmes, therefore, need to carefully review who is being reached by their services and implement interventions specifically tailored to engage people who might be missed.

There are various settings in which HTS can be provided to the public and expanded further, for instance in healthcare facilities, such as hospitals, clinics and mobile clinics, and at community sites, be these stand-alone or even home-based services, where testing services are provided within the community.^2^ There is also an option for HIV self-testing (HIVST), which is carried out by an individual who wants to know his or her HIV status and is carried out privately by the individual alone.^5^ HIV self-testing provides an opportunity for testing to be carried out discreetly and at one’s convenience, which could increase the uptake of HIV testing amongst those unable or unwilling to access other healthcare services.^2^ Concerns raised regarding HIVST include lack of HIV counselling,^30^ instructions are difficult to follow^31^ and there should be more of a focus on linkage to care.^32^

This research study has a few limitations. ‘Ever testing’ for HIV is self-reported, and therefore, prone to biases related to social desirability, recall and under-reporting. Nevertheless, the results of the nationally representative population-based survey can be generalised to adults aged 15 years and above who tested for HIV in South Africa. There may be a high degree of within-district heterogeneity. In future, work will include examining the sub-district level estimates applying the robust methodology of small area estimation, which involves using auxiliary predictors to improve the precision of imprecise district-level estimates.

## Conclusion

This study demonstrated the utility of visually displaying spatial inequities in HIV testing using nationally representative data by presenting simple maps for targeted priority setting. The findings suggest that provinces and districts with low testing coverage, especially amongst male participants, should prioritise tailored interventions to improve uptake of HIV testing. The strategies for HTS should include scaling up of HIVST and community HIV testing, specifically home-based testing to improve the uptake of HIV testing in those districts that are lagging behind in order to ensure equity in the geographical coverage of HIV testing.

## Appendix 1: Summary statistics and model output used in the secondary data analysis

**TABLE 1-A1:** Uptake of those aged 15 years and older who have ever been tested for HIV in the 52 districts of South Africa.

District name	*n*	*%*	95*%* CI
uMkhanyakude	393	54.7	45.9–63.2
Vhembe	447	61.0	56.5–65.3
Ugu	606	61.4	54.6–67.7
Sarah Baartman	450	66.2	60.7–71.4
Amathole	207	66.3	59.5–72.5
Nelson Mandela Bay	767	66.3	62.0–70.4
West Coast	306	66.6	56.5–75.4
Cape Winelands	463	67.1	60.8–72.8
Namakwa	130	67.2	51.0–80.2
Z F Mgcawu	541	67.7	61.6–73.3
iLembe	2213	68.0	62.4–73.1
uMzinyathi	1952	68.0	64.5–71.3
Zululand	303	68.8	60.4–76.2
O.R. Tambo	848	70.4	65.8–74.6
uMgungundlovu	395	70.4	59.4–79.5
uThukela	2291	70.9	67.3–74.2
Eden	239	71.1	61.2–79.3
Mopani	383	71.2	64.9–76.8
Greater Sekhukhune	791	71.8	67.4–75.7
Buffalo City	202	71.9	63.8–78.7
Xhariep	155	73.0	58.9–83.6
King Cetshwayo	2449	73.4	65.9–79.7
Alfred Nzo	173	73.5	62.8–81.9
Gert Sibande	2308	74.3	70.9–77.5
Capricorn	400	74.4	68.0–79.9
Pixley ka Seme	637	74.9	69.1–79.9
City of Cape Town	1494	75.3	72.4–78.0
eThekwini	2258	75.8	70.2–80.6
Waterberg	313	75.9	66.5–83.3
Ehlanzeni	1720	76.6	73.2–79.6
Harry Gwala	258	76.6	67.9–83.5
Dr Kenneth Kaunda	495	76.9	66.4–84.8
John Taolo Gaetsewe	169	77.4	64.5–86.6
Overberg	187	77.4	64.4–86.7
Chris Hani	152	78.2	67.4–86.1
City of Johannesburg	1123	78.2	74.8–81.3
Fezile Dabi	178	78.5	68.5–86.0
Joe Gqabi	121	78.5	75.5–81.3
Sedibeng	1838	78.8	71.3–84.8
Ekurhuleni	1289	79.0	74.9–82.7
Dr Ruth Segomotsi Mompati	231	80.3	68.1–88.6
Nkangala	813	80.4	77.0–83.4
City of Tshwane	1096	81.2	78.1–83.9
Bojanala	1458	81.3	78.6–83.7
Frances Baard	490	81.4	75.5–86.1
Central Karoo	66	81.7	70.8–89.2
Amajuba	175	83.1	72.2–90.3
Mangaung	680	83.1	78.6–86.8
West Rand	780	83.3	78.2–87.3
Thabo Mofutsanyane	491	84.8	81.3–87.8
Lejweleputswa	234	85.2	81.7–88.2
Ngaka Modiri Molema	284	86.1	79.4–90.9
**Total**	**38 442**	**75.1**	**74.1–76.0**

CI, confidence interval.

**TABLE 2-A1:** Uptake of male and female participants aged 15 years and older who have ever been tested for HIV in the 52 districts of South Africa.

District name	Male participants			Female participants		
	*n*	*%*	95*%* CI	*n*	*%*	95*%* CI
Alfred Nzo	68	58.9	43.8–72.5	105	85.4	78.7–90.2
Amajuba	63	89.9	75.5–96.3	112	78.2	66.8–86.5
Amathole	77	57.6	45.9–68.5	130	73.6	64.2–81.2
Bojanala	594	78.6	74.4–82.4	864	83.8	80.8–86.3
Buffalo City	75	64.8	55.1–73.4	127	77.3	68.0–84.5
Cape Winelands	176	68.4	61.7–74.4	287	66.0	56.6–74.3
Capricorn	141	64.6	54.0–74.0	259	81.4	77.1–85.1
Central Karoo	24	87.7	83.1–91.2	42	76.8	58.8–88.4
Chris Hani	61	75.7	57.6–87.7	91	80.4	67.1–89.2
City of Cape Town	626	70.1	66.1–73.8	868	80.5	76.9–83.7
City of Johannesburg	492	71.9	65.5–77.5	631	84.4	80.2–87.8
City of Tshwane	474	80.9	75.4–85.5	622	81.4	77.5–84.8
Dr Kenneth Kaunda	229	77.1	68.1–84.2	266	76.6	61.2–87.1
Dr Ruth Segomotsi Mompati	90	71.2	48.3–86.8	141	87.0	77.5–92.8
Eden	104	61.1	48.6–72.3	135	81.3	64.8–91.1
Ehlanzeni	709	69.4	65.0–73.5	1011	83.0	79.6–85.9
Ekurhuleni	567	74.4	68.6–79.5	722	84.0	80.3–87.1
eThekwini	933	72.8	67.7–77.3	1325	78.4	70.7–84.6
Fezile Dabi	93	73.9	61.2–83.6	85	84.3	71.5–92.0
Frances Baard	231	74.7	67.9–80.5	259	88.4	81.5–93.0
Gert Sibande	1031	69.0	64.5–73.2	1277	80.2	75.9–83.9
Greater Sekhukhune	290	62.0	55.2–68.3	501	79.1	74.0–83.3
Harry Gwala	89	69.9	60.0–78.2	169	80.9	69.0–89.0
iLembe	821	64.2	56.6–71.2	1392	71.0	66.5–75.0
Joe Gqabi	54	76.5	69.2–82.5	67	80.7	69.8–88.3
John Taolo Gaetsewe	76	73.0	56.1–85.2	93	82.0	70.3–89.7
King Cetshwayo	895	68.3	60.5–75.1	1554	77.4	69.0–84.1
Lejweleputswa	103	81.9	73.7–87.9	131	88.4	84.6–91.4
Mangaung	292	83.1	75.2–88.9	388	83.1	77.7–87.4
Mopani	162	67.2	58.9–74.5	221	74.8	67.0–81.2
Namakwa	60	68.3	45.7–84.7	70	66.1	51.7–78.0
Nelson Mandela Bay	321	62.9	56.1–69.3	446	69.5	64.4–74.2
Ngaka Modiri Molema	121	83.4	74.1–89.8	163	88.6	82.8–92.6
Nkangala	379	76.7	70.9–81.7	434	84.9	81.0–88.0
O.R. Tambo	327	59.2	53.1–65.1	521	79.6	74.9–83.7
Overberg	69	79.8	63.2–90.2	118	75.4	64.3–83.9
Pixley ka Seme	269	69.2	61.2–76.2	368	80.2	73.0–85.9
Sarah Baartman	188	63.7	57.7–69.4	262	68.6	60.0–76.0
Sedibeng	782	78.9	69.3–86.2	1056	78.7	71.2–84.7
Thabo Mofutsanyane	206	83.1	77.5–87.6	285	86.4	82.7–89.4
Ugu	254	59.3	50.4–67.7	352	63.3	54.6–71.1
uMgungundlovu	189	66.5	47.6–81.2	206	75.4	59.3–86.5
uMkhanyakude	135	48.5	37.8–59.3	258	59.0	46.6–70.3
uMzinyathi	677	62.8	57.1–68.1	1275	71.6	67.4–75.5
uThukela	812	61.8	56.2–67.1	1479	76.9	73.7–79.8
Vhembe	189	46.6	39.3–53.9	258	73.7	68.6–78.3
Waterberg	146	72.2	59.5–82.2	167	79.9	70.1–87.2
West Coast	144	58.7	46.2–70.2	162	75.8	64.0–84.6
West Rand	368	81.6	74.7–86.9	412	85.1	78.0–90.2
Xhariep	67	66.8	60.4–72.6	88	79.9	33.7–96.9
Z F Mgcawu	252	60.0	51.5–67.9	289	75.4	69.2–80.7
Zululand	126	59.9	47.8–71.0	177	76.4	70.5–81.4
**Total**	**15 721**	**70.7**	**69.4–72.0**	**22 721**	**79.2**	**78.1–80.1**

CI, confidence interval.

**TABLE 3-A1:** Excess probability of ever having tested for HIV after adjusting for age and sex in the 52 districts of South Africa.

District name	Excess probability
O.R. Tambo	0.26
Amathole	0.27
Chris Hani	0.30
Buffalo City	0.30
Sarah Baartman	0.33
Alfred Nzo	0.34
Nelson Mandela Bay	0.36
Joe Gqabi	0.41
Mangaung	0.44
Fezile Dabi	0.47
Lejweleputswa	0.48
Thabo Mofutsanyane	0.49
Xhariep	0.54
City of Johannesburg	0.36
City of Tshwane	0.41
Ekurhuleni	0.42
Sedibeng	0.43
West Rand	0.46
uMkhanyakude	0.23
uMzinyathi	0.31
Ugu	0.31
iLembe	0.32
uThukela	0.32
Zululand	0.33
Harry Gwala	0.34
uMgungundlovu	0.38
King Cetshwayo	0.38
eThekwini	0.39
Amajuba	0.50
Vhembe	0.26
Greater Sekhukhune	0.30
Capricorn	0.33
Mopani	0.34
Waterberg	0.37
Ehlanzeni	0.40
Gert Sibande	0.44
Nkangala	0.50
Dr Ruth Segomotsi Mompati	0.37
Dr Kenneth Kaunda	0.39
Ngaka Modiri Molema	0.39
Bojanala	0.42
ZF Mgcawu	0.33
John Taolo Gaetsewe	0.37
Namakwa	0.37
Pixley ka Seme	0.38
Frances Baard	0.47
Cape Winelands	0.35
City of Cape Town	0.38
West Coast	0.38
Eden	0.39
Overberg	0.40
Central Karoo	0.42

**TABLE 4-A1:** Multilevel mixed-effects logistic regression model.

Variable	OR	95% CI	*p*
Sex	1.6	1.5–1.7	< 0.001
Age	1.0	1.0–1.1	< 0.001

CI, confidence interval; OR, odds ratio.
